# Supplementary material for: Biodegradable Nanofiber/Metal–Organic Framework/Cotton Air Filtration Membranes Enabling Simultaneous Removal of Toxic Gases and Particulate Matter
Source: Polymers (Basel). 2023 Sep 30;15(19):3965. doi: 10.3390/polym15193965 (PMC10575390; doi:10.3390/polym15193965)
Supplement: Supplementary file 1 [file polymers-15-03965-s001.zip › polymers-2619385-supplementary.pdf]

Supporting Information

Biodegradable Nanofiber/Metal Organic Framework/Cotton Air Filtration Membranes Enabling Simultaneous Removal of Toxic Gases and Particulate Matter

Sujin Ryu [1,†], Doyeon Kim [1,2,†], Hyewon Lee [1], Yoonjin Kim [1], Youngbok Lee [2,3,4], Myungwoong Kim [5,*], Heedong Lee [1,*] and Hoik Lee [1,2,*]

[1] Advanced Textile R&D Department, Research Institute of Convergence Technology, Korea Institute of Industrial Technology (KITECH), 143 Hanggaulro, Sangnok-gu, Ansan 15588, Republic of Korea; sjryu@kitech.re.kr (S.R.); dobbie@kitech.re.kr (D.K.)

[2] HYU-KITECH Joint Department, Hanyang University, Ansan 15588, Republic of Korea; yblee@hanyang.ac.kr

[3] Department of Applied Chemistry, Hanyang University, Ansan 15588, Republic of Korea

[4] Department of Chemical and Molecular Engineering, Center for Bionano Intelligence Education and Research, Hanyang University, Ansan 15588, Republic of Korea

[5] Department of Chemistry and Chemical Engineering, Inha University, Incheon 22212, Republic of Korea

* Correspondence: mkim233@inha.ac.kr (M.K.); lhd0121@kitech.re.kr (H.L.); hoik@kitech.re.kr (H.L.)

† These authors contributed equally to this work.

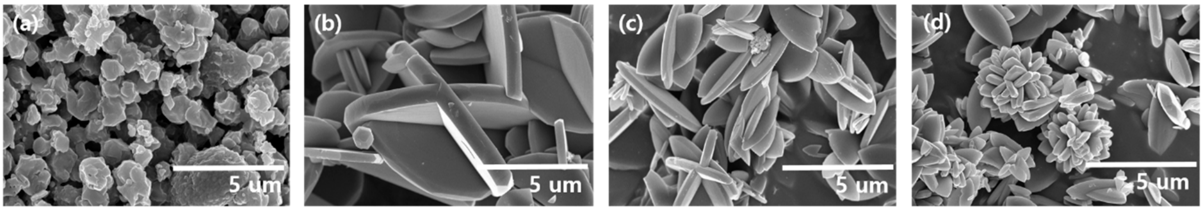

Figure S1. SEM images showing the morphology of the prepared ZIF-8 particles with different [ligand]/[Zn^{2+}] of (a) 2.0, (b) 4.0, (c) 6.0, and (d) 8.0.
